# A Case of Cavitary Lung Metastasis From Prostate Cancer

**DOI:** 10.1002/rcr2.70101

**Published:** 2025-01-24

**Authors:** Shinnosuke Ohnaka, Mayumi Aoyama, Rino Arai, Saya Hattori, Yusuke Kubo, Shugo Suzuki, Toshihiro Yoshimura, Akinori Ebihara, Toshiaki Morikawa, Hidenobu Shigemitsu, Ichiro Kuwahira

**Affiliations:** ^1^ Respiratory Disease Center Tokyo General Hospital Tokyo Japan; ^2^ Department of Urology Tokyo General Hospital Tokyo Japan; ^3^ Department of Pulmonary Medicine Tokai University Tokyo Hospital Tokyo Japan; ^4^ Department of Chest Surgery Tokyo General Hospital Tokyo Japan; ^5^ Department of Critical Care Medicine St. Rose Dominican Siena Hospital Las Vegas Nevada USA

**Keywords:** cavitary lung metastasis, FDG‐PET, necrosis, prostate cancer, thoracoscopy

## Abstract

A 79‐year‐old man was found to have multiple nodules in the lung fields on chest computed tomography. Metastatic lung cancer was suspected; however, the primary site remained elusive. After 1 year of follow‐up, both the nodules had enlarged. After 2 years, one of the nodules continued to enlarge; however, the other nodule cavitated and decreased in size. Concomitantly, the previously observed fluorodeoxyglucose uptake in the cavitated nodules disappeared. A comprehensive search for the primary cancer included a thoracoscopic lung biopsy which revealed that these nodules were metastatic lung lesions from prostate cancer. Pathological examination revealed necrosis within these metastatic lesions. To date, only three case reports of cavitary lung metastases from prostate cancer have been published; however, no explanation has been provided for the pathological mechanism of cavitation. To our knowledge, this is the first case to provide a potential explanation for cavitation in metastatic lung lesions from prostate cancer.

## Graphical abstract

To date, only three case reports of cavitary lung metastases from prostate cancer have been published; however, no explanation has been provided for the pathological mechanism of cavitation. We performed a thoracoscopic lung biopsy. This is the first case to provide a potential explanation for cavitation in these lesions.

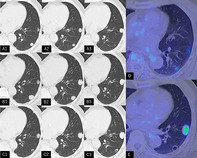

## 1 Introduction

The lungs are the second most common site of prostate cancer metastasis in autopsy cases, although the frequency of diagnosis of lung metastasis clinically based on imaging findings is very low, ranging from 3.6% to 8.0% [1, 2]. In addition, lung metastases are often accompanied by metastases to multiple organs, including the bones and intra‐abdominal lymph nodes. Furthermore, prostate cancer with metastasis only to the lungs is extremely rare, accounting for less than 5% of all cases [3]. Only three cases of cavitary lung metastases from prostate cancer have been reported in the literature; however, the mechanism of cavitation remains elusive. In our case, some of the metastatic lung nodules from prostate cancer cavitated and decreased in size. We believe that our report is the first to demonstrate the mechanism of cavitation in these metastatic lung nodules through extensive pathological evaluation of lung tissue obtained by thoracoscopic partial lung resection.

## 2 Case Report

**FIGURE 1:**
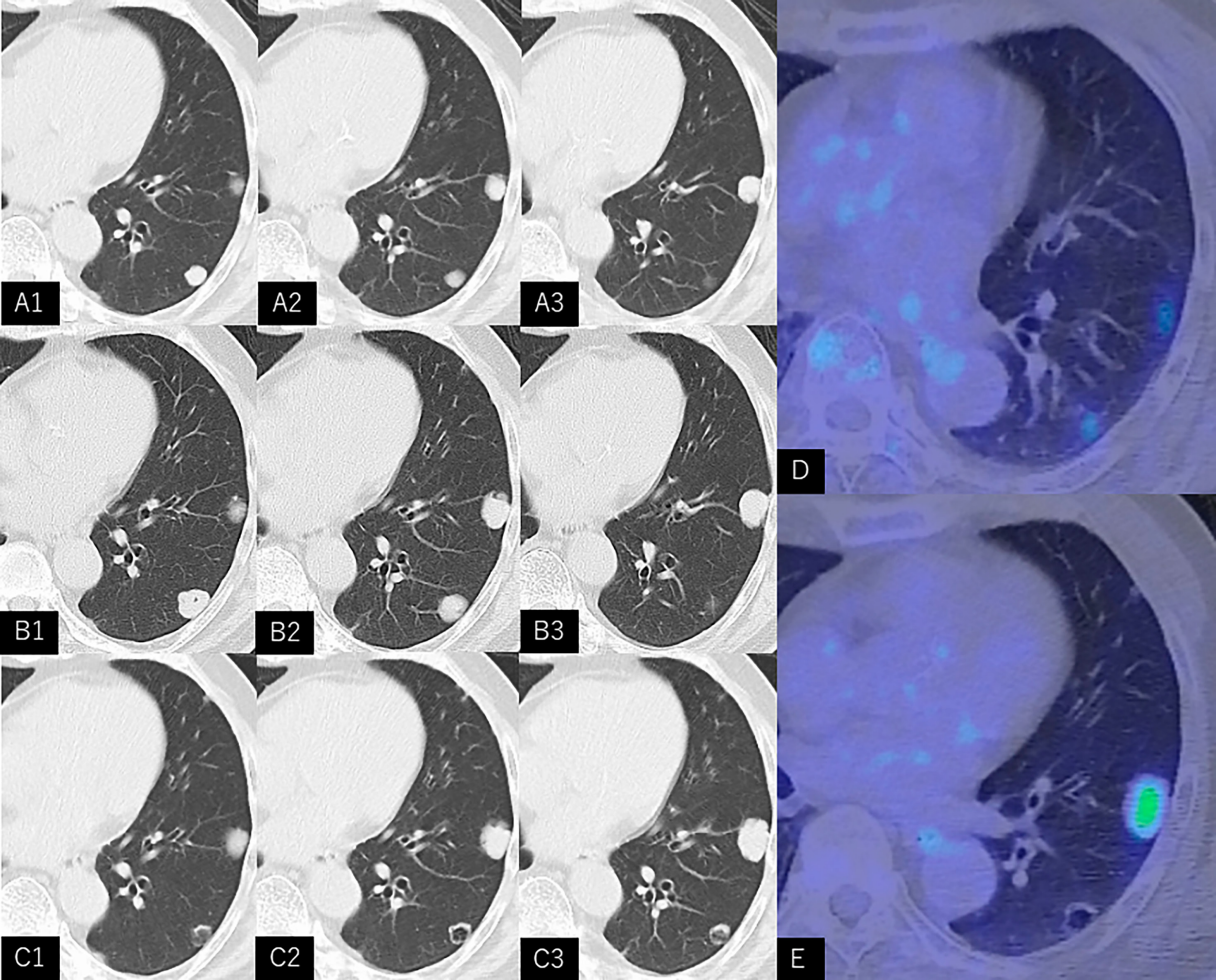
Chest computed tomography (CT) scan (A1–A3, B1–B3, and C1–C3) and fluorodeoxyglucose‐ positron emission tomography (FDG‐PET) findings (D, E). Two years before referral to our institution, a chest CT revealed nodular opacities in S6 and S8 of the left lower lobe (A1–A3). A PET scan showed FDG uptake within the nodules (SUVmax 2.34 for S6 and 2.40 for S8) (D). A follow‐up chest CT scan after 1 year showed that both nodules were enlarged (B1–B3). A chest CT scan after 2 years showed that the S8 nodule had enlarged in size; however, the S6 nodule had cavitated and decreased in size (C1–C3). A PET scan showed FDG uptake in the S8 nodule (SUVmax 4.26) but not in the S6 nodule (E).

A 79‐year‐old man was referred to our hospital with abnormal chest radiographic findings. The patient was a non‐smoker with a history of hypertension. Two years before his referral, he underwent a comprehensive evaluation for abnormal opacities noted on chest radiographs during his annual health checkup. Subsequent chest computed tomography (CT) scan revealed nodules in S6 and S8 of the left lower lobe (Figure 1A1–A3). This was followed by a positron emission tomography (PET) scan that revealed positive fluorodeoxyglucose (FDG) uptake (SUVmax 2.34 for S6 and 2.40 for S8) within these nodules that were suggestive of a lung tumour (Figure 1D). These nodules were suspected to be metastatic lesions, and no other sites with abnormal FDG uptake were observed on the PET scan. The patient did not have any symptoms, and basic blood laboratory tests were normal. Subsequently, an evaluation for the primary cancer was initiated, including upper and lower gastrointestinal endoscopy which did not reveal anything abnormal. However, the patient did not want further evaluation and was discharged.

**FIGURE 2:**
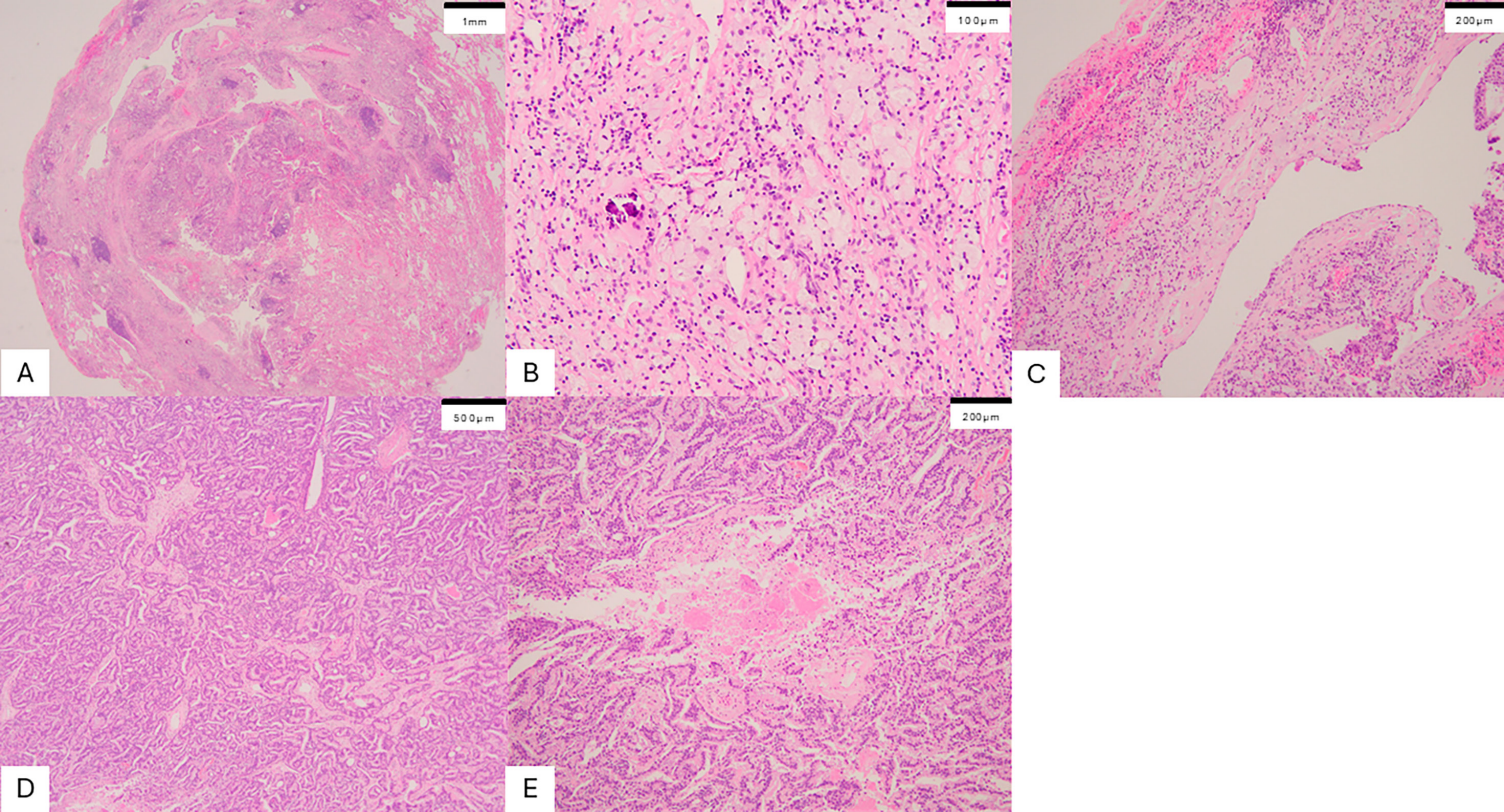
Pathological findings of the S6 (A–C) and the S8 lesions (D, E) excised under thoracoscopy. Both specimens showed adenocarcinoma. The nodule in S6 had adenocarcinoma in part, surrounded by necrotic tissue or areas of tissue loss (haematoxylin and eosin, A 20×, B 200×). Figure C shows a magnified image of the cavity wall and the surrounding tissue loss area (haematoxylin and eosin, 100×). The cavity wall shown on the left side of the figure is part of the pleura. It is thought that this tissue loss area originally occupied a much larger space, as seen on CT, but was reduced by deformation during removal from the thorax. The nodule in S8 was mostly an adenocarcinoma (haematoxylin and eosin, D 40×), but focal areas of necrosis were observed in the magnified image (haematoxylin and eosin, E 100×).

A follow‐up chest CT scan after 1 year showed that both nodules had enlarged (Figure 1B1–B3). The patient remained asymptomatic and did not wish to undergo further evaluation. Subsequently, a chest CT scan after 2 years showed that the left S8 nodule had further increased in size, whereas the left S6 nodule had decreased in size with cavitation (Figure 1C1–C3). A PET scan showed FDG uptake in the S8 nodule (SUVmax 4.26) and left iliac lymph node (SUVmax 3.57), but no uptake in the left S6 nodule (Figure 1E). No other FDG uptake was observed in the prostate. We determined that these CT findings were atypical of metastatic lung tumours. The patient consented to further evaluation and underwent a partial thoracoscopic pulmonary resection to confirm the aetiology of the nodular lesions. Figure 2A–C, show pathological findings of the S6 lesion, whereas Figure 2D,E show that of the S8 lesion. Examination revealed that the S6 nodule was mostly necrotic tissue; however, both the S6 and S8 lesions had tumour components with glandular lumen formation, suggesting pulmonary metastasis from prostate cancer. The cavity wall shown on the left side of Figure 2C is part of the pleura. It is thought that the tissue loss area on the right side of Figure 2C originally occupied a much larger space, as seen on CT, but was reduced by deformation during removal from the thorax.

As previously mentioned, FDG uptake was observed in the left iliac lymph node, which led to a closer examination of the prostate. Subsequently, elevated prostate‐specific antigen (PSA) levels (44.5 ng/mL) were noted. Diffusion‐weighted pelvic magnetic resonance imaging (MRI) revealed diffusion restriction in the right lobe of the prostatic apex and an irregular low‐signal area in the same region on T2‐weighted images. Thereafter, a prostate biopsy revealed tumour components similar to those seen in the lung metastases, leading to the diagnosis of prostate cancer, a Gleason Score of 4 + 4 = 8, rT2aN1M1. The patient is currently receiving treatment with Degarelix and the PSA level has decreased to 2.48 ng/mL.

## 3 Discussion

The frequency of lung metastasis from prostate cancer that can be clinically diagnosed based on imaging findings is very low, ranging from 3.6% to 8.0% [1, 2]. Lung metastases are often accompanied by metastases to multiple organs, including the bones and intra‐abdominal lymph nodes. Prostate cancer with no metastatic sites other than the lungs is extremely rare, accounting for < 5% of cases [3].

Metastatic lung tumours usually grow in size, however, in the present case, one of the metastases cavitated and decreased in size. To date, there have been three case reports of lung metastases from prostate cancer with cavitation. However, none of these case reports have included pathological investigations of the cavitation mechanism. In our case, considering the difficulty of the bronchoscopic approach in accurately reaching the lung nodules, and the possibility of pneumothorax and seeding by needle biopsy, these nodules were excised via partial thoracoscopic pulmonary resection. As a result, a thorough pathological examination of the entire tissue that included these nodules was performed. The S6 lesion, which had cavitated and decreased in size, was mostly necrotic. However, phagocytosis from histiocytes was observed in the necrotic tissue, suggesting that active necrosis had progressed, decreasing the lesion size. The mechanism of cavitation is thought to involve the expulsion of necrotic tissue from the airway. In contrast, the S8 lesion, which increased in size, predominantly contained tumour components, although progressive necrosis was observed in some lesions. To date, the mechanism of cavitation within metastatic lung nodules from prostate cancer had been unknown; however, the present case provides a possible explanation. The previous three cases that reported cavitary lung metastases from prostate cancer and this present case share the characteristic that the only site of distant metastases was the lungs. However, the mechanisms underlying the absence of metastases to other organs remain unknown. Further accumulation of similar cases is required to elucidate the exact mechanism. In this case, the patient was followed up at another hospital where PSA was not evaluated. After referring him to our hospital, PSA was examined and found to be high. It is possible that his PSA was elevated 2 years ago if it had been checked according to the guidelines [4]. PSA levels should be evaluated in patients with an unknown primary cancer [4].

Although it has been reported that 4% of lung metastases from malignant tumours show cavitation [5], our case demonstrated that metastatic lesions from prostate cancer also cavitate due to necrosis and, as a result, decrease in size. Prostate cancer should be considered in the differential diagnosis of the primary cancer site when searching for pulmonary cavitary nodules.

## Author Contributions

Conception or design of the work, the acquisition, analysis or interpretation of data for the work: Shinnosuke Ohnaka, Mayumi Aoyama, Rino Arai, Saya Hattori, Yusuke Kubo, Shugo Suzuki, Toshihiro Yoshimura, and Ichiro Kuwahira. Drafting the work or reviewing it critically for important intellectual content: Shinnosuke Ohnaka, Akinori Ebihara, Toshiaki Morikawa, Hidenobu Shigemitsu, and Ichiro Kuwahira. Final approval of the version to be published: Shinnosuke Ohnaka, Mayumi Aoyama, Rino Arai, Saya Hattori, Yusuke Kubo, Shugo Suzuki, Toshihiro Yoshimura, Akinori Ebihara, Toshiaki Morikawa, Hidenobu Shigemitsu, and Ichiro Kuwahira.

## Ethics Statement

The authors declare that appropriate written informed consent was obtained for the publication of this manuscript and accompanying images.

## Conflicts of Interest

The authors declare no conflicts of interest.

## Data Availability

The data that support the findings of this study are available from the corresponding author upon reasonable request.
